# Human immunodeficiency virus exposed child feeding and maternal enriching factors

**DOI:** 10.1017/S204867902400051X

**Published:** 2024-09-25

**Authors:** Birhan Desalegn, Misgan Legesse, Fassikaw Kebede Bizuneh

**Affiliations:** 1 North Wollo Zone Health Office, Woldia, North East Ethiopia; 2 College of Health Science, Woldia University, Woldia, North East Ethiopia; 3 College of Medicine and Health Science, Debre Markos University, North West Ethiopia

**Keywords:** Ethiopia, Exposed infant, Infant feeding, Mothers, Virus

## Abstract

Globally, each year 1.3 million neonates acquire human immunodeficiency virus during pregnancy, labour, and breastfeeding time. Replacing breastfeeding with recommended safe infant feeding practices significantly reduces the risk of transmission, nearly eliminating it. This study aimed to assess Human immunodeficiency virus exposed child feeding among 314 mothers with infants under 24 months old. Participants were selected using a systematic random sampling technique, and data were collected through a semi-structured questionnaire. Bivariable and multivariable logistic regression analyses employed to identify determinants for safe infant feeding. During interviews, the mean age of women was 32.35 years (standard deviation±4.5), and infants were 10.8(±3.951) months. The overall safe infant feeding was 67.2% (95% CI: 61.7, 72.9), with a mean knowledge score. By the study’s end, 9 infants (2.89%) were confirmed to be infected with virus based on dried blood sample test. Maternal promoting factors for safe infant practice included infant age 25–35 years (adjusted odd ratio (aOR) =2.9) completing high school education (adjusted odd ratio = 9.2), having a good knowledge score for infant feeding (adjusted odd ratio = 8.2), and urban residency (adjusted odd ratio = 2.2). On the other hand, being married made it 83% less likely for safe infant feeding practices (adjusted odd ratio = 0.17) compared to those never in a union. Two in three mothers practiced safe infant feeding for their HIV-exposed infants, with a mean knowledge score of 70.3%. Therefore, healthcare providers give accurate information and counselling services to make informed decisions about infant safe feeding.

## Introduction

Mother-to-child HIV transmission is the primary mode of infection for infants during pregnancy, birth, or breastfeeding.^(1)^ Mothers living with human immunodeficiency virus can strive always to breastfeed with negative outcomes for their health and the health of their kids.^(2)^ However, the mixed feeding practices for HIV-infected mothers increase the risk of HIV transmission by 3-4-fold.^(2)^ Reducing this transmission is a critical global public health challenge faced by researchers, healthcare professionals, policymakers, and HIV-infected women worldwide.^(3,4)^

Globally, around 36.7 million people, primarily in sub-Saharan Africa (71%), are living with HIV and each year, 600,000 infants globally are infected with HIV, averaging 1,600 infections per day.^(5,6)^ Nearly half (42.5%) were infected during pregnancy, labour, and breastfeeding, especially where mixed feeding is predominant in sub-Saharan Africa, strain of economic burden causes postnatal transmission.^(2,3,7)^

In low- and middle-income countries (LMICs), WHO advises HIV-infected mothers on combined antiretroviral therapy (cART) to breastfeed infants for 12–24 months, supported by the heightened risks of morbidity and mortality in formula-fed babies due to infections and malnutrition.^(8)^ Limited access to clean water and the high cost of formula milk in impoverished populations underscore the importance of this recommendation.^(9,10)^ Maternal knowledge of proper newborn safe feeding procedures, including when and how to start, significantly affected the transmission rate of HIV.^(6,11)^ The 2016 Ethiopian Demographic and Health Survey disclosed significant HIV-related insights, with 1.5 million new cases and 680,000 reported deaths, and notably 74% of Ethiopian women are aware of HIV transmission through breast milk.^(2,6,11)^

Previous systematic reviews^(1,12,13)^ and primary studies^(3,4,14–19)^ have highlighted key factors, including CD4 count, viral load, and ART adherence influencing the prevention of HIV transmission. The Ethiopian government promotes infant health and HIV-free survival through safe infant feeding in Option B+ care for all pregnant women.^(19)^ However, in 2016 an Ethiopia Demographic and Health Survey (EDHS) reported, that children had low rates of dietary diversity (4.3%) and meal frequency (47.7%), with 17% practicing safe infant feeding for all HIV-exposed infants.^(4,11,14,17,20,21)^ In Ethiopia, as of the updated guidelines in 2018, the Prevention of Mother-To-Child Transmission (PMTCT) guidelines recommend breastfeeding as the safest option for HIV-positive mothers, particularly those who have achieved high viral load suppression. However, it is important to note that the gradual introduction of mixed feeding is highly advised for lactating women in such cases.^(1,22)^ However, several economic and peer support challenges caused a significant risk of HIV transmission with a 3.54% to 4.23% rate practiced before 6 months of mixed feeding.^(3,6)^

Previous research suggests that various maternal factors impact on PMTCT to infants including maternal education (12 instances),^(21)^ employment status (7 instances),^(23)^ maternal knowledge, and attitude (5.9 to 15.2 instances),^(23)^ HIV disclosure status (6.2 times) were identified as significant hindrances to achieving zero transmission.^(25)^ Therefore, this study aimed to estimate levels of HIV-exposed infants’ safe feeding practices and maternal enriching factors in Northeast Ethiopia.

## Methods

### Study area and period

The study was conducted between April 1 and June 20, 2023, in the North Wollo zone, Amhara region, Northeast Ethiopia. The zone is centred on Woldia and is located 521 km from Addis Ababa and 360 km from the regional capital, Bahir Dar. It shares borders with the South Gondar zone in the West, the South Wollo zone in the South, the Afar region in the East, the Tigray region in the Northeast, and the Waghimra zone in the North West sides. The projected population for 2023 was estimated at 1,763,246, with 50.2% females and 13% residing in urban areas. The zone consists of 14 districts, including three town administrative areas. Healthcare facilities in the zone include 6 public hospitals, 69 health centres, 309 health posts, 10 private medium clinics, 42 primary clinics, and 33 pharmacies. Among these, five hospitals and 22 health centres provide ART services.^(31)^

### Study design

A multi-centre, institution-based, descriptive cross-sectional study was conducted among 314 HIV-infected mothers with their dyads less than 24 months.

### Source population

All mothers attending antiretroviral therapy (ART) services with infants ≤24 months under Option B+ care in selected health institutions form the sampled population.

#### Inclusion criteria

From April to June 2023, all HIV-infected women who had infants less than 24 months of age and were receiving treatment at the ART unit were eligible for this study.

#### Exclusion criteria

Mothers who were severely ill and unable to communicate their HIV status, as well as their children’s HIV status, were excluded from the study

### Sample size determinations

The sample size was determined using the single population proportion formula using a 95% confidence level and a 5% margin of error, as well as the prevalence of infant feeding practice, which was found to be 75.2%. The formula used to calculate the sample size is as follows: n = (Zα/2) ^2^ [p (1−p)]/d². Where: n = required sample size Zα/2 = critical value for the normal distribution at a 95% confidence interval, which is equal to 1.96 p = prevalence (75.2%), d = margin of error (5%). Using the given values, the calculation for the sample size is as follows: n = (1.96)² [0.752(1-0.752)]/(0.05)², which results in n = 286. However, after accounting for a non-response rate of 10%, the sample size is adjusted by non-response rate by adding10% non-response rate as 286 + (0.10 × 286) = 314. Hence, the final sample size was found to be 314 HIV-infected mothers with their dyads were interviewed for final analysis.

### Sampling procedure

In the North Wollo zone, there were 27 health facilities providing Option B+ services (PMTCT), comprising 5 public hospitals and 22 health centres. To select the sampled participants, 30% of the facilities were first randomly selected from the total 27 Option B+ services giving centres by using a lottery method. This resulted in a sample of eight health facilities for final sample size selection based on their serving-giving population over the past 3 months. In each health facility, a 3-month file of service was given population divided by our sample size, and we determined K =, then we selected a total of 314, using systematic sampling technique within each facility using the formula (K = N/n, 837/314 = 3) where N represents the total client population (837) and n denotes the required sample size (314). The final sample of 314 participants was selected using a k = 3 interval from each health facility based on their population level.

#### Dependent variable

This study evaluated WHO-recommended infant feeding practices (Yes/No), emphasising safety for up to 2 years. Unsafe practices include early breastfeeding substitution, introducing complementary foods at 6 months or earlier, or mixed feeding before 6 months. In LMICs, WHO recommends 12–24 months of breastfeeding for HIV-infected mothers cART, and gradual introduction of complementary feeding with stressing strict adherence to ART prophylaxis given for infants.^(1,3,16)^

#### Independent variable

Maternal Age, Marital status, Occupation, Educational status, Monthly income, Knowledge of vertical transmission, Disclosure of HIV status, Place of delivery, Antenatal follow-up, Stage of HIV, CD4 Count, Breast problem, Counseling practice, Sex of the child, Age of child

### Operational definition

#### Minimum dietary diversity

The Children’s Complementary Food Dietary Diversity Score (DDS) was based on seven food groups: grains/roots/tubers, legumes/nuts, dairy products, flesh foods (meats/fish/poultry), eggs, vitamin A-rich fruits and vegetables (VAFV), and other fruits and vegetables (OFV). The DDS, ranging from 0 to 7, measured dietary diversity by assigning one point to each food group. We considered minimum dietary diversity as consuming food from at least four different groups (DDS ≥ 4).^(24)^ **Exclusive breastfeeding;** Exclusive breastfeeding involves giving only maternal breast milk to the infant for the first 6 months, while replacement feeding entails providing a diet with all necessary nutrients for infants not receiving any breast milk. Mixed feeding occurs when infants under 6 months are given liquids or foods alongside breast milk.^(1)^ **Complementary feeding;** HIV-exposed infants require careful feeding, following guidelines for exclusive breastfeeding for the first 6 months, followed by the introduction of complementary foods while breastfeeding up to 12 months. ART is crucial in reducing HIV transmission risk, and decisions on infant feeding should involve healthcare providers, considering specific circumstances and ongoing monitoring for the well-being of these infants during the transition to complementary feeding.^(25,26)^ **Mean Good knowledge;** Good knowledge is defined as respondents who scored equal to or greater than the mean score of maternal safe infant feeding related knowledge question list in WHO guidelines.^(26,27)^

#### Data collection procedure and quality control

The questionnaire, initially in English, was translated into Amharic. Six data collectors and three supervisors underwent a 1-day training on study objectives, privacy, and confidentiality. Data collection used a pre-tested semi-structured questionnaire, with a pilot study conducted on a 5% sample size for adjustments. Daily supervision by supervisors and the principal investigator ensured consistency, completeness, clarity, and accuracy in the data collection process.

#### Data process and analysis

Collected data underwent editing, entry, and coding with EPI info v7.2.5.0 software, then analysed using SPSS v25. Results were presented through frequency tables, figures, and percentages. Maternal knowledge of infant feeding, assessed with nine structured questions, produced a mean score. Bivariate and multivariable logistic regressions were performed with independent variables having P-value<0.25 in bivariate analysis. Collinearity effects and data normality were checked, by applying a stepwise backward elimination procedure. Categorical variables with adjusted odds ratios and 95% confidence intervals determined safe infant feeding at P<0.05. Model fitness was assessed using the Hosmer–Lemeshow goodness-of-fit test. Maternal knowledge was categorised as poor or good based on the mean score, and the study questions were adapted from a previously published article.^(4,6,11,14,17,22,24,28–30)^ Cronbach’s alpha yielded reliability coefficients of 0.76 for maternal knowledge and 0.82 for infant feeding practices, signifying good internal consistency. Tables containing all maternal knowledge and safe infant feeding-related questions were incorporated into the study.^(3,5,14,15,17,23,31,32)^

## Result

### Socio-demographic characteristics

The study included 314 HIV-infected women with infants from eight public health institutions, achieving a 100% response rate. The mean age for mothers and children was 32.4 years (SD±4.4) and 7.8 months (SD±2.9), respectively. Among live-birth infants, 63.7% were female, and 36.6% were male. The majority of participants (72.3%) lived with their spouses, and 38.2% had no formal education. Urban residents accounted for 60.2%, with 73.6% being housewives. The majority (90.3%) completed their fourth antenatal care (ANC) visit, while 4.6% had a history of home delivery. Additionally, 270 pregnant women had a CD4 count of ≤50 cells/mm3.

### Maternal and obstetrics characteristics

Nearly half of the 165 respondents (52.5%) had fewer than two children, and the majority of the 304 participant women (96.8%) received counselling on infant feeding options. Among the mothers who gave birth, 283 (90.1%) delivered at health institutions, with 277 (88.2%) having a spontaneous vaginal delivery. During ANC care, 142 women (45.2%) became aware of their HIV status, while 139 (44.3%) knew about their pregnancy before initiating ANC. Additionally, 210 respondent mothers (66.9%) disclosed their HIV status (Table 1).

**Table 1.** Socio-demographic characteristics of HIV-positive mothers and their infants attending ART service

Variable	Category	Frequency	Percent (%)
Maternal Age	<25	7	2.2
	25-30	98	31.2
	31-35	128	40.8
	> = 36	81	25.8
Residency	Rural	125	39.8
	Urban	189	60.2
Age of the child	<6 month	48	15.3
	>6 month	266	84.7
Sex of the child	Male	115	36.6
	Female	199	63.4
Marital status	Married	227	72.3
	Divorce	53	16.9
	Widowed	34	10.8
Maternal education	Unable to read and write	120	38.2
	Primary school	85	27.1
	High school	61	19.4
	Diploma and above	48	15.3
Occupation status	Housewife	231	73.6
	Government employee	31	9.9
	Private employee	6	1.9
	Daily labourer	1	0.3
	Merchant	24	7.6
	Farmer	21	6.7
Maternal anti-natal care	Yes	283	90.1
	No	31	9.6
Maternal post-natal care	Yes	59	18.8
	No	255	81.2
Counselling for infant feeding	Yes	304	96.8
	No	10	3.2
Place of birth	Health institution	300	95.5
	Home delivery	14	4.6
Mode of Delivery	SVD	277	88.2
	CS	37	11.8
Attending post-natal care	Yes	141	44.9
	No	153	55.1
HIV Disclosed status	Friends	7	3.2
	Husband	307	96.9
Maternal CD4 count (cell/mm3)	≤500 cell/mm3	40	12.7
	>500 cell/mm3	274	83.3
Disease progress	Stage 1	66	21.0
	Stage 2	146	46.5
	Stage 3	102	32.5
	Stage 4	0	0
Ever encountered a breast problem	Yes	101	32.16
	No	213	67.8
If yes Type of breast problem (N = 101)	Burning, tingling-	5	4.9
	Cracked nipples	18	17.8
	Engorgement	59	58.4
	Sore nipples	19	18.8
long term illness	Yes	42	13.4
	No	272	86.6

### Maternal knowledge for safe infant feeding practice

Over half of 165 respondents (52.5%) had <2 children, and 96.8% of 304 women received feeding counselling. For those giving birth, 90.1% delivered at health institutions, with 88.2% having spontaneous vaginal delivery. During interviews, mothers mentioned HIV transmission: 63.37% (200) during delivery, 7.9% (24) during breastfeeding, and 32.4% (97) during breast pain, oral ulcers of infants, and mother problems (Table 2).

**Table 2.** Maternal knowledge of infant feeding practice among HIV-positive women attending ART service

Variables	Categories	Frequency	Percent
1. When can HIV be transmitted from mother to child	During breastfeeding	200	63.7
	During delivery	24	7.9
	During breast pain, oral ulcer of infanta, and mother’s problem	97	32.9
2. At which age stage were you informed to start infant complementary?	Before 6 months advisable after birth	94	29.9
	Exactly at 6 months’ completion	177	56.4
	After 12 months of only breastfeeding	47	13.7
3. Which is best Prevention means from mother-to-child Transmission (MTCT) after birth	Maternal Good ART adherence with breastfeeding	146	46.5
	Giving ART to the child as prophylaxis	131	41.7
	Taking ART drugs during pregnancy	17	5.5
	Using infant formula feeding after birth	20	9.9
4. Infant Feeding outweighed breastfeeding after birth	Not sure/ don’t know	23	7.3
	Yes	291	92.7
5. Do you know infants have acquired HIV through mixed feeding before?	Yes	297	94.6
	No /Not Sure	17	5.4
6. Does breastfeeding outweigh that infant-feeding option?	Yes	289	92.8
	No/Not Sure	25	9.1
7. What are the advantages and disadvantages of using expressed and heat-treated breast milk	The HIV in breast milk is inactivated by heating and yet most of the nutrients are preserved.	159	50.6
	Breast milk is the perfect food for babies even if it has risk	155	49.4
8. How long should exclusive breastfeeding be recommended during counseling by healthcare providers	Up to 6 months	60	19.1
	Beyond 6 months	131	41.7
9. Mothers with HIV should receive evidence-based, patient-centred counselling for decision-making about infant feeding before delivery	No	3	0.99
	Yes	311	98.1
10. Do you know that mixed feeding children can cause HIV	Yes	305	97.1
	No/ I don’t know	9	2.9
11. Healthcare providers discuss on risk of mixed infant feeding	Yes	298	94.9
	No/ don’t remember	16	5.01
12. Maternal Knowledge score	Good	183	70.3
	Poor	131	39.7

### Maternal practice for safe infant feeding

Almost all (99%) of mothers received safe infant feeding demonstrations and counselling during ANC from healthcare providers. During the interview, 203 (64.49%) of them also practiced demonstrated how breastfeeding after the discussion (Table 3).

**Table 3.** Maternal practicing related questions for HIV exposed infant feeding

WHO recommendation for safe infant feeding question	Categories	Frequency	Percent
1. Have you practiced Infant Feeding Demonstrations after healthcare providers showed	No/I don’t remember	1	0.3
	Yes	313	99.7
2. Infant Age informed for safe infant feeding (n = 313)	During ANC follow-up	94	29.9
	At delivery ward	177	56.4
	After being delivered at PNC	46	13.01
3. Which option do you want to practice with your infant (n = 314)	Mixed method with prophylaxis from infant after birth	3	0.8%
	Exclusive breastfeeding with prophylaxis ceased following complementary feeding.	311	99.01%
3.1 Reasons for EBF with prophylaxis ceased upon complementary feeding (n = 311)	Advised by parents and friends:	24	7.6
	Counselled by health professionals	63	20.1
	Fear of HIV transmission: 129 (40.1%)	129	
	Fear of stigma	48	15.3
	Norm of society	12	3.8
	Lack of information	18	5.7
	Aware of the safety of the baby	17	5.4
	Advised by parents and friends:	27	8.6
4. Breastfeeding the baby at the time of the interview	No	203	64.6
	Yes	111	35.4

### Mothers’ status during an interview

During the study, 67.2% of mothers practiced safe infant feeding, with a mean knowledge score of 70.3%. The majority of mothers (83.3%) had a CD4 count greater than 500 cells/mm3, and 146 (46.5%) were classified as WHO clinical stage II. Among the infants, 81.2% had not experienced any oral ulcers.

### Level of safe infant feeding practice

The overall prevalence of safe infant feeding practices was 67.2% (95% CI: 61.7, 72.9), whereas the remaining 32.8% of participants used mixed or unsafe infant feeding options. Among women who used unsafe infant feeding options mainly reported having breast problems with (n = 101), the most common breast problem reported was engorgement (58.4%), followed by sore nipples 19(18.8%), cracked nipples 18(17.8%), and burning or tingling 5(4.9%).

### Factors affecting infant feeding practice

During the final multivariable logistic regression of this report, variables with a P-value<0.25 on bivariate analysis were considered candidates for multivariable regression. These included mother and infant age, residence, marital status, education, number of children, ANC visits, place of delivery, timing of breastfeeding, maternal knowledge score of infant feeding practices, HIV disease progression, presence of long-term illness, and infant mouth ulcers.

After controlling certain confounding factors, five variables were significantly associated with safe infant feeding during the PMTCT. These include being maternal age with 25–35 years (adjusted odd ratio (aOR) = 2.9, 95% CI: 1.2, 7.6), completing high school education (aOR = 9.2, 95% CI: 1.3, 6.8), having a good knowledge score for infant feeding (aOR = 8.2, 95% CI: 2.1, 32.7), and urban residency (aOR = 2.2, 95% CI: 1.1, 4.5) are maternal enriching factors as compared with their respective counter groups. On the other hand, infant mothers living with their spouses had an 83% reduced likelihood of safe infant feeding practices compared to those who were never in a union (aOR = 0.17, 95% CI: 0.36, 0.80) but having baby mothers (Table 4).

**Table 4.** Factors affecting infant feeding practices among HIV-positive mothers attending ART service

Variable	Category	Infant feeding practice		95% CI for COR	95% CI for aOR	P value
		Safe	Unsafe			
Age of the infant	≤6 month	42(13.5)	6(1.92%)	Reference	Reference	
	>6 month	169(53.8%)	97(30.8%)	1.56(1.14, 4.72)	1.23(0.98, 1.67)	0.057
Infant age	12-24	84 (79.4%)	20(20.6%)	5.80(2.7, 10.3)	0.68(0.3, 1.6)	0.39
	6-18	93(73.1%)	36(27.9%)	3.57(1.9, 6.4)	4.1(1.2, 13.1)	0.026*
	>35	34(42%)	47(58%)	Reference	Reference	
Marital status	Married	161(70.9%)	66(29%)	1.17(0.3, 5.7)	0.17(0.4, 0.8)	0.02*
	Divorce	34(61.2%)	19(38.8%)	2.0(0.8, 4.8)	0.8(0.4, 1.2)	0.09
	Widowed	16(47.1%)	18(52.9%)	Reference	Reference	
Residency	Rural	106(84.8%)	19(15.2%)	Reference	Reference	
	Urban	105(55.5%)	84(44.5%)	4.46(2.5, 7.8)	2.2(1.3, 4.5)	
Source of information	Health institution	208(69.1%)	89(29.9%)	10.9(3.7, 13.8)	5.2(2.3, 16)	0.01*
	Family and friends	3(17.7%)	14(82.3%)	Reference	Reference	
Maternal knowledge	Good mean score	204(69.2%)	91(30.8%)	Reference	Reference	
	Poor mean score	7(36.8%)	12(63.2%)	12.2(3.1, 16)	8.2(2.4, 17.8)	0.003*

* Indicated statistical significant variables after association.

## Discussion

At the end of the study periods, the overall safe infant feeding practice among mothers for their dyads was found to be 67.2%). This finding is consistent with previously reported 63.43% in Gondor Hospital,^(14)^ 63.8% in Samra Hospital,^(6)^ 63.99% in Debre Markos,^(11)^ and 63.8% in Bahir Dar Hospital.^(32)^ This might be related to healthcare providers using similar guidelines for therapeutics, and counselling principles across different study settings similar contextual factors, such as cultural norms, available resources, and healthcare policies, may have influenced safe infant feeding practices across the included healthcare facilities. Conversely, the final report of safe infant feeding practices is higher than previously found at 25.5% in Gondar Hospital,^(24)^ 49.3% in Wolaita Soda Hospital,^(2)^ and 18.2% in Kenya Hospital,^(15)^ but lower than previously reported 86.4% in Gondar hospital.^(4)^ These differences may result from variations in maternal healthcare utilisation across different Ethiopian facilities and the discount could stem from differences in study settings, access to information, and technology, influencing community awareness. Maternal workload and limited time for childcare may contribute to these disparities.

The final report of this study indicated that the mean maternal knowledge score on infant feeding was found to be 70.3%, which is lower compared to previous reports of 86.4% and 65.3% in,^(28)^ 50.1% found in Botswana,^(23)^ 83.6% in Nigeria.^(30)^ This indicates the existence of gaps in mother-to-child transmission and the actual practice in Ethiopia and the healthcare providers sometimes struggle to influence maternal behavioural changes in implementing recommended infant feeding choices which is an urgent need for better counselling for pregnant women on infant feeding options to eliminate transmission.

Regarding maternal enriching factors for safe infant feeding factors identified, accordingly, mothers of HIV-exposed infants within 30–35 years of age were two-fold times more likely to adopt safe infant feeding practices compared with counter groups. The findings of this study are consistent with previous findings in Gondar town,^(16)^ SNNPR regions,^(28)^ Amhara region,^(33)^ Northern Kenya,^(19)^ and southern Nigeria.^(34)^ The possible reasons for the similarity might be due to middle-aged mothers who had exposed infants are more likely to adhere to any recommended medical practice

Consistent with previous study findings in Gondar referral hospital,^(16)^ North America, and Nigeria,^(34)^ married women with HIV-exposed infants were 83% less likely to adopt the safe way of feeding practices compared to divorced women. The possible explanation may be that married women may face unsupportive partners upon disclosing their HIV status, affecting the adoption of safe feeding practices. HIV disclosing can lead to social challenges, including from husbands, friends, and community members when transitioning from exclusive breastfeeding.

The finding of the current study also indicated that getting information or counselling about safe infant feeding from the correct health professionals was significantly associated with adopting the recommended infant feeding practices that counter group. This is consistent with the study done in Addis Ababa,^(35)^ Woldia town,^(21)^ and Oromia regions.^(30)^ This could be because many women find that receiving skilled information on infant feeding options may not be enough for informed decision-making, and it helps them choose appropriate feeding methods, improve adherence, and opt for safer options like exclusive breastfeeding or complete avoidance of breastfeeding.^(1)^

Consistent with study previous studies done in the Amhara region^(33)^ and Addis Abeba City,^(36)^ mothers with good knowledge about safe infant feeding are more likely to practice it. This might be related to preventing mother-to-child transmission and promoting safe infant feeding, early adoption is encouraged. HIV-positive women need customised counselling to make informed choices about feeding options based on local circumstances, ensuring optimal growth for their babies. Moreover, the findings of this study reveal that individual women with permanent urban residency were 2.2 times more likely to adopt safe infant feeding practices compared to rural dwellers. This finding aligns with previous studies conducted in Gondar town^(16)^ and Nigeria.^(9)^ The possible reason is that the majority of urban residents had previous exposure, and those in urban areas tend to be more receptive to the training and guidance provided by medical professionals. They exhibit an eagerness to acquire and apply information, which may contribute to their higher adoption of safe infant feeding practices.

### Limitations of the study

This cross-sectional study design limited the ability to establish a cause-and-effect relationship, and there was a possibility of recall bias as mothers were expected to remember the feeding patterns of their children since birth.

### Conclusion

This study’s findings have important implications for public health interventions targeting infant feeding practices among HIV-positive mothers. The majority (67.2%) followed safe guidelines, but mixed feeding (32.8%) increased HIV transmission risk. Predictors included age, marital status, residency, access to information, and knowledge of recommended options and targeted interventions through healthcare providers give accurate information and counselling services, for mothers to make informed decisions about infant feeding.

## References

[1] Dagnew AB , Teferi MD. A systematic review and meta-analysis on adoption of WHO-recommended infant feeding practices among HIV-positive mothers in Ethiopia. BMC Pregnancy Childbirth. 2021;21(1):194. 33685405 10.1186/s12884-021-03662-3 PMC7941701

[2] Daniel Baza AA , Markos M. Infant feeding practices among HIV positive mothers enrolled in selected public health institutions of Wolaita, Ethiopia: facility-based multicenter cross-sectional study. PAMJ - One Health. 2022;7(38). 10.11604/pamj-oh.2022.7.38.33580.

[3] Ejara D , Mulualem D , Gebremedhin S. Inappropriate infant feeding practices of HIV-positive mothers attending PMTCT services in Oromia regional state, Ethiopia: a cross-sectional study. Int Breastfeed J. 2018;13:37. 30140299 10.1186/s13006-018-0181-x PMC6098608

[4] Genetu H , Yenit MK , Tariku A. Breastfeeding counseling and support are associated with continuous exclusive breastfeeding from one week to six months of age among HIV exposed infants in north Gondar zone, Ethiopia: a cross-sectional study. Int Breastfeed J. 2016;12:21. 28439291 10.1186/s13006-017-0113-1 PMC5401345

[5] Mutawulira I , Nakachwa J , Muharabu L , Wilson Walekhwa A , Kayina V. Exploring infant feeding practices and associated factors among HIV-positive mothers attending early infant diagnosis clinic in Northern Uganda. Epidemiol Infect. 2022;150:e130. 10.1017/S0950268822001091 PMC9306008 35718949

[6] Zewdu D , Bekele DM , Bantigen KA , Wake AD. Unsafe Infant feeding practice and associated factors among HIV positive mothers attending PMTCT in Ethiopia: a cross-sectional study. HIV AIDS (Auckl). 2023;15:325–337. 37342283 10.2147/HIV.S414636 PMC10277203

[7] Ekubagewargies DT , Mekonnen HS , Siyoum TM. Assessment of knowledge, attitude, and practice of HIV positive mothers on antiretroviral treatment towards infant feeding in Gondar Town Health Institutions, North West Ethiopia, 2017. Int J Pediatr. 2019;2019:9107989. 30713565 10.1155/2019/9107989 PMC6332993

[8] Abuogi L , Noble L , Smith C , Committee On P , Adolescent HIV , Section On B. Infant feeding for persons living with and at risk for HIV in the United States: clinical report. Pediatrics. 2024;153(6):e2024066843. 38766700 10.1542/peds.2024-066843

[9] Fassinou LC , Songwa Nkeunang D , Delvaux T , Nagot N , Kirakoya-Samadoulougou F. Adherence to option B + antiretroviral therapy and associated factors in pregnant and breastfeeding women in Sub-Saharan Africa: a systematic review and meta-analysis. BMC Public Health. 2024;24(1):94. 38183014 10.1186/s12889-023-17004-9 PMC10768427

[10] Bansaccal N , Van der Linden D , Marot J-C , Belkhir L. HIV-Infected mothers who decide to breastfeed their infants under close supervision in Belgium: about two cases. Front Pediatr. 2020;8:248. 32537442 10.3389/fped.2020.00248 PMC7266974

[11] Temesgen H , Negesse A , Getaneh T , et al. Feeding practices among human immunodeficiency virus-exposed infants in Ethiopia: systematic review and meta-analysis. Adv Public Health. 2021;2021:1–12.

[12] Endalamaw A , Demsie A , Eshetie S , Habtewold TD. A systematic review and meta-analysis of vertical transmission route of HIV in Ethiopia. BMC Infect Dis. 2018;18(1):283. 10.1186/s12879-018-3189-3. PMC6013937 29929480

[13] Kassa GM. Mother-to-child transmission of HIV infection and its associated factors in Ethiopia: a systematic review and meta-analysis. BMC Infect Dis. 2018;18:216. 10.1186/s12879-018-3126-5. 29747581 PMC5946547

[14] Belay GM , Wubneh CA. Infant feeding practices of HIV Positive mothers and its association with counseling and HIV disclosure status in Ethiopia: a systematic review and meta-analysis. Hindawi AIDS Res Treat. 2019;2019(1):Article ID 3862098, 13 pages. 10.1155/2019/3862098. PMC6699255 31467708

[15] Andare N , Ochola S , Chege P. Determinants of infant feeding practices among mothers living with HIV attending prevention of mother to child transmission Clinic at Kiambu Level 4 hospital, Kenya: a cross-sectional study. Nutr J. 2019;18(1):64. 31677638 10.1186/s12937-019-0490-y PMC6825715

[16] Sendo EG , Mequanint FT , Sebsibie G. Infant feeding practice and associated factors among HIV positive mothers attending ART clinic in governmental health institutions of Bahir Dar Town, Amhara Regional State, Ethiopia, 2017. J AIDS Clin Res. 2018;9:1. 10.4172/2155-6113.1000755.

[17] Muluye D , Woldeyohannes D , Gizachew M , Tiruneh M. Infant feeding practice and associated factors of HIV positive mothers attending prevention of mother to child transmission and antiretroviral therapy clinics in Gondar Town health institutions, Northwest Ethiopia. BMC Public Health. 2012;12:240. 22449092 10.1186/1471-2458-12-240 PMC3326701

[18] Tchakoute CT , Sainani KL , Osawe S , et al. Breastfeeding mitigates the effects of maternal HIV on infant infectious morbidity in the Option B+ era. AIDS (London, England). 2018;32(16):2383–2391. 30134300 10.1097/QAD.0000000000001974

[19] Yapa HM , Drayne R , Klein N , et al. Infant feeding knowledge and practice vary by maternal HIV status: a nested cohort study in rural South Africa. Int Breastfeed J. 2020;15(1):77. 32873311 10.1186/s13006-020-00317-5 PMC7466779

[20] Astewaya M , Tirhas T , Tessema B. Assessment of factors associated with infant and young child feeding practices of human immunodeficiency virus (HIV) positive mothers in selected hospitals of Southern Nations, Nationalities, and Peoples Region (SNNPR) Ethiopia. J AIDS HIV Res. 2016;8(6):80–92.

[21] Girma M , Wendaferash R , Shibru H , Berhane Y , Hoelscher M , Kroidl A. Uptake and performance of prevention of mother-to-child transmission and early infant diagnosis in pregnant HIV-infected women and their exposed infants at seven health centers in Addis Ababa, Ethiopia. Trop Med Int Health: TM & IH. 2017;22(6):765–75. 28407452 10.1111/tmi.12881

[22] Kebede F , Kebede T . Incidence and predictors of attrition rate after children started inpatient treatments for complicated severe acute malnutrition in North West Ethiopia. J Health Popul Nutr. 2022;41(1):54. 10.1186/s41043-022-00332-8. 36447294 PMC9706866

[23] Ndubuka J , Ndubuka N , Li Y , Marshall CM , Ehiri J. Knowledge, attitudes and practices regarding infant feeding among HIV-infected pregnant women in Gaborone, Botswana: a cross-sectional survey. BMJ Open. 2013;3(11):e003749. 10.1136/bmjopen-2013-003749 PMC3845062 24293206

[24] Esubalew F , Atenafu A , Abebe Z. Feeding practices according to the WHO-recommendations for HIV exposed children in northwest Ethiopia: a cross-sectional study. Clin Nutr ESPEN. 2018;28:114–120. 10.1016/j.clnesp.2018.08.019. 30390866

[25] Misganaw A , Naghavi M , Walker A , et al. Progress in health among regions of Ethiopia, 1990–2019: a subnational country analysis for the Global Burden of Disease Study 2019. The Lancet. 2022;399(10332):1322–1335. 10.1016/S0140-6736(21)02868-3 PMC8987934 35294898

[26] WHO. Guideline Updates on HIV and Infant Feeding. Accessed 1 January 2016. Published 2016. https://wwwwhoint/publications/i/item/9789241549707.

[27] WHO. HIV-Infant-Feeding-Revised-Principles-Recommendations-Rapid-Advice Guideline. Accessed 1 January 2016. Published 2009. https://wwwaidsdatahuborg/sites/default/files/resource/hiv-infant-feeding-revised-principles-recommendations-rapid-advicepdf.

[28] Bekere A , Garoma W , Beyene F. Exclusive breastfeeding knowledge of HIV positive mothers and associated factors in selected health institutions of West Oromia, Ethiopia. Univ J Food Nutr Sci. 2014;2(3):37–44.

[29] Hazemba AN , Ncama BP , Sithole SL. Promotion of exclusive breastfeeding among HIV-positive mothers: an exploratory qualitative study. Int Breastfeed J. 2016;11:9. 27103938 10.1186/s13006-016-0068-7 PMC4839145

[30] Umeobieri AK , Mbachu C , Uzochukwu BSC , et al. Perception and practice of breastfeeding among HIV positive mothers receiving care for prevention of mother to child transmission in South-East, Nigeria. Int Breastfeed J. 2018;13:50. 30519275 10.1186/s13006-018-0191-8 PMC6264606

[31] Lang’at PC , Ogada I , Steenbeek A , et al. Infant feeding practices among HIV-exposed infants less than 6 months of age in Bomet County, Kenya: an in-depth qualitative study of feeding choices. Arch Dis Childhood. 2018;103(5):470–473. 29317437 10.1136/archdischild-2017-314521

[32] Naturinda R , Akello G , Muwonge C , et al. Mothers’ knowledge and practice on modified infant feeding for prevention of postnatal HIV transmission in post-conflict northern Uganda district. Int J Infect Dis. 2016;45:267.

